# Dietary Fucoxanthin Increases Metabolic Rate and Upregulated mRNA Expressions of the PGC-1alpha Network, Mitochondrial Biogenesis and Fusion Genes in White Adipose Tissues of Mice

**DOI:** 10.3390/md12020964

**Published:** 2014-02-14

**Authors:** Meng-Ting Wu, Hong-Nong Chou, Ching-jang Huang

**Affiliations:** 1 Department of Biochemical Science and Technology, National Taiwan University, No. 1, Sec. 4, Roosevelt Road, Taipei 10617, Taiwan; E-Mail: dreamstop@gmail.com; 2 Institute of Fisheries Science, National Taiwan University, No. 1, Sec. 4, Roosevelt Road, Taipei 10617, Taiwan; E-Mail: unijohn@ntu.edu.tw

**Keywords:** fucoxanthin, adipose tissue, metabolic rate, PGC-1α network, mitochondrial biogenesis and fusion

## Abstract

The mechanism for how fucoxanthin (FX) suppressed adipose accumulation is unclear. We aim to investigate the effects of FX on metabolic rate and expressions of genes related to thermogenesis, mitochondria biogenesis and homeostasis. Using a 2 × 2 factorial design, four groups of mice were respectively fed a high sucrose (50% sucrose) or a high-fat diet (23% butter + 7% soybean oil) supplemented with or without 0.2% FX. FX significantly increased oxygen consumption and carbon dioxide production and reduced white adipose tissue (WAT) mass. The mRNA expressions of peroxisome proliferator-activated receptor (PPAR) γ coactivator-1α (PGC-1α), cell death-inducing DFFA-like effecter a (CIDEA), PPARα, PPARγ, estrogen-related receptor α (ERRα), β3-adrenergic receptor (β3-AR) and deiodinase 2 (Dio2) were significantly upregulated in inguinal WAT (iWAT) and epididymal WAT (eWAT) by FX. Mitochondrial biogenic genes, nuclear respiratory factor 1 (NRF1) and NRF2, were increased in eWAT by FX. Noticeably, FX upregulated genes of mitochondrial fusion, mitofusin 1 (Mfn1), Mfn2 and optic atrophy 1 (OPA1), but not mitochondrial fission, Fission 1, in both iWAT and eWAT. In conclusion, dietary FX enhanced the metabolic rate and lowered adipose mass irrespective of the diet. These were associated with upregulated genes of the PGC-1α network and mitochondrial fusion in eWAT and iWAT.

## 1. Introduction

Obesity, defined as excess accumulation of adipose, is a worldwide endemic health problem. Obesity and its related disorders are associated with increased morbidity, mortality and healthcare costs [1]. Mitochondria play an important role in adipose biology [2]. Mitochondria dysfunction is linked to obesity [3] and type 2 diabetes [4,5]. Mitochondria biogenesis is thus considered a potential target for the intervention of insulin resistance in obesity and diabetes [6,7,8]. Mitochondrion is a dynamic organelle that is continuously remodeled by fusion and fission [9], which is important for bioenergetic adaptation to metabolic demand. Cells exposed to a nutrient-rich environment tend to maintain their mitochondria in a separated state, while cells under starvation tend to have mitochondria in a connected state [10]. Reduction in the mitochondrial network, but unaltered mitochondrial mass have been reported in skeletal muscle of obese Zucker rats [3] and type 2 diabetic patients [11]. Moreover, mRNA and the protein, Mfn2, an important protein located in the mitochondrial outer membrane and mediating mitochondrial fusion, were reduced in skeletal muscle of obese subjects compared to lean subjects [12].

Peroxisome proliferator-activated receptor γ coactivator-1α (PGC-1α) is a strong promoter of mitochondrial biogenesis and oxidative metabolism through nuclear respiratory factor, NRF1 and NRF2, ERRα, PPARγ and PPARα [13,14,15]. ERRα further activated the transcriptional activity of the Mfn2 promoter, and the effects were synergic with those of PGC-1α [16]. In addition, NRF1 and NRF2 themselves are able to stimulate mitochondrial transcription factor A (TFAM), a mitochondrial matrix protein essential for the replication and transcription of mitochondria DNA [17,18]. PGC-1α mRNA expression is reduced in subcutaneous fat of morbidly obese patients [19]. Ectopic expression of PGC-1α in white adipose tissue (WAT) increased brown adipocyte specific genes, including uncoupling protein 1 (UCP1) and mitochondrial activity [20]. 

Fucoxanthin (FX) is a major carotenoid in brown algae and has an unusual allenic structure [21]. FX has a suppressive effect on adipose accumulation in genetically diabetic KK-*A*^y^ mice [22,23,24], Wistar rats [25,26] and diet-induced obese C57BL/6J mice [27,28,29]. FX feeding significantly increased fecal triglyceride and cholesterol excretion and β-oxidation in liver and epididymal WAT (eWAT) and also reduced fatty acid, cholesterol synthesis-related enzyme activity, serum and hepatic lipid accumulation [28,30,31]. Previous studies also showed that FX increased UCP1 mRNA expression in abdominal WAT of mice with reduced adipose mass [25,28,31]. PGC-1α expression in the skeletal muscles was upregulated in KK-*A*^y^ mice fed FX [32].

UCP1 is a thermogenic mitochondria protein that was thought to express exclusively in brown adipose tissue (BAT). However, its expression in WAT has been confirmed in most recent studies, and these UCP1 expressing adipocytes in WAT were named “Beige/Brite” or “recruited (in contrast to the traditional “constituted”) brown adipocytes [33,34]. Increases in the recruited brown adipocytes can enhance thermogenic activity and reduce adiposity in rodents [33,34] and humans [35]. As a dominant regulator of mitochondrial biogenesis and oxidative metabolism [15], PGC-1α regulates thermogenic activity by inducing the expression of UCP1 and key enzymes of the mitochondrial respiratory chain in both constituted and recruited brown adipocytes [33]. Mitochondrial biogenesis and dynamics regulate mitochondrial function, respiratory capacity, apoptosis and oxidative phosphorylation and, thus, impact energy balance [3,12,36]. Whether FX increased expressions of genes in the PGC-1α regulated pathways in WAT and metabolic rate has not been reported. 

Here, we examined the suppressive effect of adipose accumulation of FX under a high sucrose or a high saturated fat diet using a 2 × 2 factorial design. Whole body O_2_ consumption and CO_2_ production were measured and expressions of PGC-1α regulated genes in WAT analyzed.

## 2. Results and Discussion

### 2.1. FX Decreased White Adipose Weight without Altering Food Intake

Four groups of mice were respectively fed the 4 test diets shown in Table 1. The results of two-way ANOVA on the four groups (Table 2) of mice showed that neither FX nor diet affected final body weight, body weight gain and energy efficiency (*p* > 0.05). The high fat diet, but not FX, decreased food and energy intake (*p* < 0.05) (Table 2). FX significantly decreased relative weight of both abdominal, eWAT and retroperitoneal WAT (rWAT), and subcutaneous iWAT (*p* < 0.001). High fat diet decreased, but FX increased, the relative weight of BAT, (*p* < 0.05) (Table 3). Liver, kidney, spleen and heart relative weights were significantly increased by FX (Supplementary Table S1).

**Table 1:** Composition of the 4 test diets: HS, high sucrose diet; HS + F, high sucrose diet supplemented with fucoxanthin; HF, high fat diet; HF + F, high fat diet supplemented with fucoxanthin. The composition of vitamin mix and mineral mix are in accordance with AIN-93 and AIN-93G, respectively [37].

Ingredients of Diet (g/kg)	HS	HS + F	HF	HF + F
Corn starch	129.5	129.5	209.35	209.35
Sucrose	500	500	100	100
Butter	−	−	230	228
Soybean oil	70	68	70	70
Casein	200	200	260	260
Cellulose	50	50	65	65
AIN-93 vitamin mix	10	10	13	13
AIN-93G mineral mix	35	35	45.5	45.5
l-Cystine	3	3	3.9	3.9
Choline bitartrate	2.5	2.5	3.25	3.25
Fucoxanthin	−	2	−	2

**Table 2:** Initial body weight, final body weight, body weight gain, food/energy intake and energy efficiency of C57BL/6J male mice fed test diets for five weeks. Values are the means ± SD (*n* = 4). * denotes significant influence by either dietary factor at *p* < 0.05 analyzed by two-way ANOVA. Energy efficiency = grams of body weight gain/1000 kcal energy intake. FX, fucoxanthin.

Dietary groups	Initial body weight	Final body weight	Body weight gain	Food intake	Energy intake	Energy efficiency
	g	g	g	g/day	kcal/day	
HS	19.27 ± 1.06	24.19 ± 1.75	4.92 ± 0.76	3.12 ± 0.39	12.37 ± 1.53	11.42 ± 1.66
HS + F	19.24 ± 0.58	24.63 ± 1.03	5.39 ± 0.59	3.16 ± 0.20	12.45 ± 0.78	12.35 ± 0.92
HF	19.28 ± 0.69	24.27 ± 1.71	4.99 ± 1.31	2.29 ± 0.14	11.44 ± 0.72	12.45 ± 3.01
HF + F	19.21 ± 0.61	24.84 ± 1.26	5.63 ± 0.98	2.27 ± 0.06	11.28 ± 0.30	14.28 ± 2.67
	*p* values					
Diet	0.9769	0.8469	0.7474	<0001 *	0.0460 *	0.2085
FX	0.9001	0.5050	0.2656	0.9331	0.9391	0.2381
Diet * FX	0.9717	0.9257	0.8628	0.7604	0.8075	0.6943

**Table 3:** Relative tissue weights (percent of body weight) of C57BL/6J male mice fed test diets for five weeks. Values are the means ± SD (*n* = 4). * denotes significant influence by either dietary factor at *p* < 0.05 analyzed by two-way ANOVA. iWAT, inguinal white adipose tissue (WAT); eWAT, epididymal WAT; rWAT, retroperitoneal WAT; BAT, brown adipose tissue.

Group	HS	HS+F	HF	HF + F	*p*-values		
					Diet	FX	Diet * FX
iWAT	0.56 ± 0.13	0.39 ± 0.03	0.63 ± 0.08	0.34 ± 0.08	0.8149	0.0002 *	0.1599
eWAT	1.77 ± 0.48	1.10 ± 0.12	1.90 ± 0.42	1.21 ± 0.21	0.3944	0.0006 *	0.9527
rWAT	0.40 ± 0.18	0.13 ± 0.04	0.44 ± 0.24	0.14 ± 0.06	0.7321	0.0003 *	0.9982
BAT	0.29 ± 0.05	0.37 ± 0.04	0.26 ± 0.03	0.30 ± 0.02	0.0198 *	0.0066 *	0.3840

### 2.2. FX Enhanced Metabolic Rate

**Figure 1:**
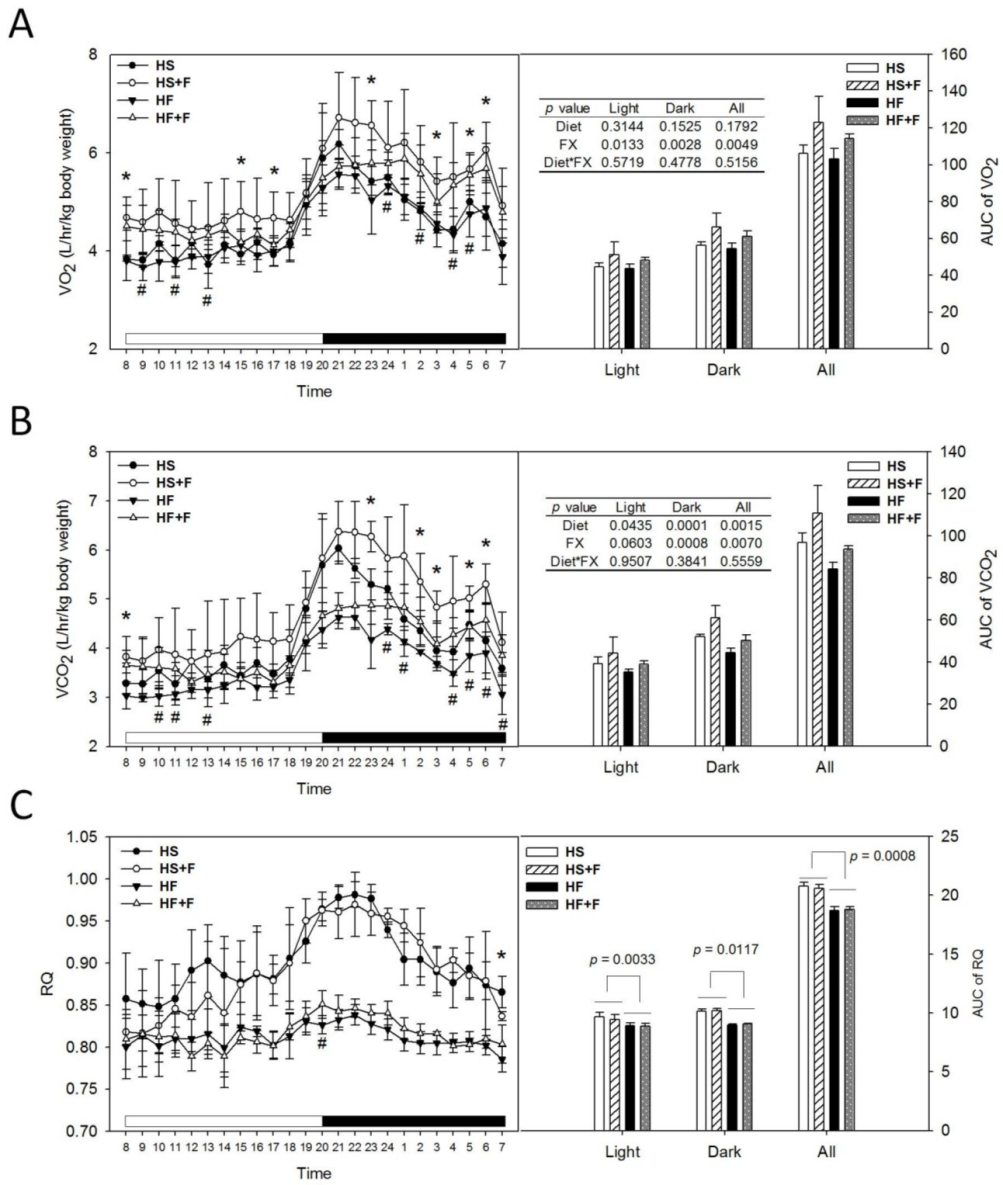
O_2_ consumption (**A**), CO_ 2_ production (**B**), respiratory quotient (**C**) and their area under the curve (AUC) of test mice at week 3. Mice were individually placed in metabolic chambers with free access to water and their respective diet and monitored for six days. Values are means and error bars are SD (*n* = 4). * denotes significant difference between groups HS and HS + F, *p* < 0.05; # denotes significant difference between groups HF and HF + F, *p* < 0.05, analyzed by the Student’s *t*-test. AUCs of O_2_ consumption (VO_2_), CO_2_ production (VCO_2_) were analyzed by two-way ANOVA. The AUC of RQ was analyzed by the Wilcoxon rank-sum test.

Mice were placed in respiratory chambers at week 3 for six days to continuously monitor O_2_ consumption (VO_2_), CO_2_ production (VCO_2_). These and calculated respiratory quotients (RQ) values throughout a day and respective area under the curve (AUC) values were shown in Figure 1. At seven out of 24 hour time points, high sucrose diet supplemented with fucoxanthin (HS + F) or high-fat diet (HF + F) groups had significantly higher VO_2_ and VCO_2_, respectively, compared to HS and HF groups (Figure 1A,B). Diet did not change the AUC of VO_2_ (Figure 1A), but the high fat diet decreased that of the VCO_2_ (Figure 1B). FX increased the AUC of both VO_2_ and VCO_2_ through the dark phase and all day without an interaction with the diet. Mice fed high fat diets had significantly lower AUC of RQ (*p* < 0.05), indicating that these mice used more fat as their energy source. FX supplementation did not change RQ (Figure 1C), implying that FX might have enhanced the common aerobic metabolic pathway of glucose and fat metabolism. Indeed, FX was shown to enhance the utilization of glucose in skeletal muscle [32] and liver [29], as well as fatty acid β-oxidation in WAT [28] and liver [30].

**Figure 2:**
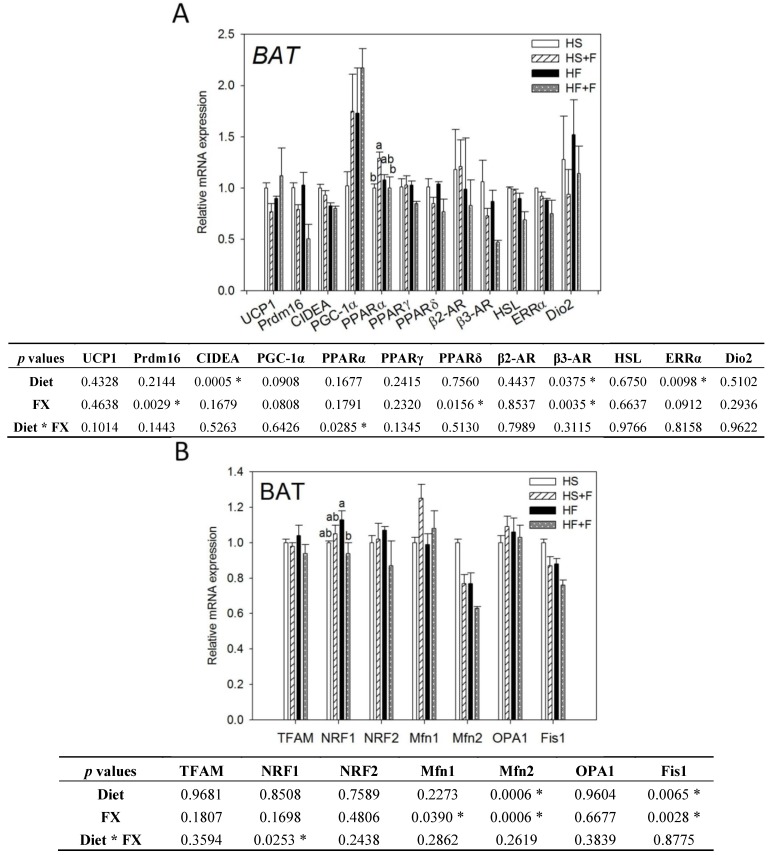
Thermogenic (**A**) and mitochondrial homeostasis-related (**B**) mRNA levels in brown adipose tissue of mice fed test diets for five weeks. Values are means, and error bars are SEM (*n* = 4). * denotes significant effect by either dietary factor at *p* < 0.05 analyzed by two-way ANOVA. When an interaction (*p* < 0.05) between diet and FX existed, the significance of differences among the HS, HS + F, HF and HF + F groups was further analyzed by Duncan’s multiple range test. HS, HS + F, HF and HF + F: As indicated in Figure 1. Relative mRNA expression was measured by real-time qRT-PCR using β-actin as the internal control and normalized to group HS.

### 2.3. Effect of FX on Thermogenic and Mitochondrial Homeostasis-Related Gene Expressions in BAT and Serum Hormone Concentration

Both high fat diet and FX lowered β3-AR, Mfn2 and Fis1 mRNA levels in the BAT (*p* < 0.05). Mice fed high fat diets had significantly lower CIDEA and ERRα mRNA expression in the BAT (*p* < 0.05). FX also decreased Prdm16 and PPARδ but increased Mfn1 mRNA in the BAT. Neither diet nor FX changed PGC-1α UCP1, PPARα, PPARγ, β2-AR, HSL, Dio2, TFAM, NRF1, NRF2 and OPA1 mRNA expressions in BAT. Among them, diet and FX had an interaction effect on the PPARα and NRF1 mRNA expression (*p* < 0.05). FX increased PPARα mRNA expression in the high sucrose diet-fed mice, but not in the high fat diet-fed mice. FX reduced NRF1 mRNA expression in the high fat diet-fed mice, but not in the high sucrose diet-fed mice (Figure 2). 

In this study, BAT mass was significantly increased by FX as in some of previous reports [23,24,25], although other studies did not observe such an effect [31,38]. This discrepancy might be associated with the different mouse strain, gender and diet formula used. Although BAT mass was increased by FX and positively correlated to carbon dioxide production (*p* = 0.02) during the dark period (Supplementary Figure S1), we did not observe any thermogenic genes upregulated in BAT. β3-AR, Prdm16 and PPARδ mRNA were even downregulated by FX. To validate these results, we even conducted the Housekeeping Genes PCR array (Qiagen, Germantown, MD, USA) and employed three popular software packages (GeNorm, NormFinder and BestKeeper) to confirm the use of β-actin as the most stable housekeeping gene. 

On the other hand, our serum hormone analysis showed that the norepinephrine concentration was decreased by FX (Table 4). Norepinephrine, the sympathetic neurotransmitter, is the main *in vivo* stimulator of the adrenergic signaling mediating the BAT thermogenic machinery. It is not known whether this low serum norepinephrine in FX-fed mice is related to the very minor change in the BAT gene expression irrespective of the enlarged mass observed in this study. Moreover, no significant correlation between BAT mass and O_2_ consumption was observed (Supplementary Figure S1). Taken together, it is speculated that BAT contributes little, if any, to the FX enhanced O_2_ consumption. 

**Table 4:** Serum thyroid hormone, (nor)epinephrine and corticosterone concentrations of C57BL/6J male mice fed test diets for five weeks. Values are the means ± SD (*n* = 4). * denotes a significant effect by either dietary factor at *p* < 0.05 analyzed by two-way ANOVA. T4, thyroxine; T3, triiodothyronine; NE, norepinephrine; E, epinephrine; Cort, corticosterone.

	HS	HS + F	HF	HF + F	*p*-values		
					Diet	FX	Diet * FX
T4, nM	24.59 ± 3.69	20.73 ± 5.22	18.94 ± 2.88	17.55 ± 3.79	0.0467 *	0.2117	0.5464
T3, nM	0.80 ± 0.21	0.87 ± 0.34	0.80 ± 0.14	0.82 ± 0.21	0.8297	0.6998	0.8432
T4/T3	32.70 ± 11.11	27.42 ± 14.18	23.80 ± 1.92	21.62 ± 2.66	0.1346	0.4312	0.7415
NE, nM	92.26 ± 16.52	66.75 ± 11.69	105.72 ± 20.01	72.05 ± 10.17	0.2383	0.0021 *	0.5989
E, nM	3.05 ± 0.75	3.52 ± 1.21	5.61 ± 3.09	4.98 ± 3.03	0.0949	0.9931	0.5768
NE/E	31.87 ± 10.69	20.62 ± 7.69	22.09 ± 7.96	17.53 ± 6.75	0.1512	0.0843	0.4413
Cort, ng/mL	71.11 ± 10.15	63.99 ± 20.76	55.55 ± 32.38	58.69 ± 23.35	0.3898	0.8384	0.6634

### 2.4. FX Induced Thermogenic-Related Gene Expressions in eWAT and iWAT

**Figure 3:**
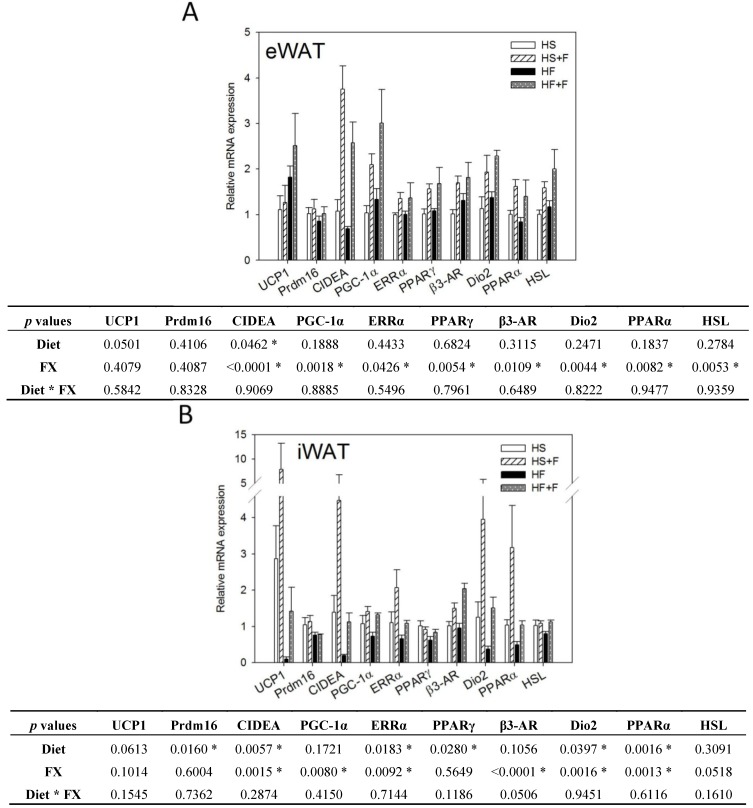
Thermogenic gene expressions in epididymal (**A**) and inguinal (**B**) white adipose tissue. Values are means, and error bars are SEM (*n* = 4). * denotes significant effect by either dietary factor at *p* < 0.05 analyzed by two-way ANOVA. HS, HS + F, HF and HF + F: as indicated in Figure 1. The measurement of relative mRNA expression is as indicated in Figure 2.

While the high fat diet decreased CIDEA mRNA expression, FX significantly increased mRNA expressions of CIDEA, PGC-1α, ERRα, PPARγ, β3-AR, Dio2, PPARα and HSL in eWAT (Figure 3A). The high fat diet decreased UCP1, CIDEA, Prdm16, ERRα, PPARγ, Dio2 and PPARα mRNA expression, but FX increased CIDEA, PGC-1α, ERRα, β3-AR, Dio2 and PPARα mRNA expression in iWAT (Figure 3B). FX did not significantly affect mRNA expressions of these genes in the abdominal rWAT (data not shown), except for upregulating PGC-1α.

Expressions of UCP1, Prdm16, CIDEA and PGC-1α in WAT are indicators for the “browning” of WAT, which could reduce the adverse effects of WAT and help to improve metabolic health [34,39]. In this study, we observed increases in the mRNA of CIDEA and PGC-1α, but not UCP1 and Prdm16 in eWAT and iWAT in mice fed the FX diets. The expression of UCP1 is transcriptionally regulated by the adrenergic signaling (β-AR, cAMP-dependent protein kinase A (PKA), *etc.*) coupled to PGC-1α and PPAR [40]. PGC-1α has been shown to be required for exercise-induced UCP1 expression in WAT [41]. Prdm16, by co-activating PGC-1α increased mitochondrial content, as well as enhanced uncoupled respiration. Prdm16 activates a robust brown fat phenotype, including the induction of PGC-1α, UCP1 and Dio2 [42,43,44]. In this study, although UCP1 mRNA in these WATs of FX-fed mice was slightly higher than that of the respective controls, the difference did not reach statistical significance, due to the low number of animals per group (*n* = 4), as well as the large individual variations. Like our study, Woo *et al.* did not observe increased UCP1 in eWAT of mice fed the 0.2% FX diet, although mice fed the 0.05% FX diet did show an increase of UCP1 in eWAT [31]. CIDEA has been shown to inhibit uncoupling activity of UCP1 when co-expressed in yeast and increases mitochondrial coupling by suppressing UCP1 expression [45]. Therefore, the unchanged UCP1 mRNA level in this study could also be related to the dose of FX and the elevated CIDEA mRNA expression in WAT. Induction of CIDEA has been used as a marker for the emergence of brown adipocyte-like cells in WAT [46]. As we found that only two of the four “beige” adipocyte-specific genes were upregulated, whether FX induces the whole program of WAT “browning” cannot be confirmed. 

On the other hand, we observed that β3-AR, Dio2, PGC-1α, ERRα and PPARα mRNA in both eWAT and iWAT and HSL in eWAT were elevated by FX. The adrenergic signaling (β3-AR, PKA, *etc.*) coupled to PGC-1α and PPAR regulates UCP1 expression [40]. PGC-1α also controls mitochondria biogenesis and respiratory function through targeting multiple transcription factors, like NRF1, NRF2 and ERRα. β3-AR is expressed abundantly and predominantly in brown and white adipocytes. Treatment of mice with β3-AR agonists increases oxygen consumption [47]. Under β3-AR stimulation, PKA is activated and further turns on the transcription of Dio2 and PGC-1α and phosphorylation of HSL [48,49]. Dio2 catalyzes the conversion of T4 to T3 (the active form of thyroid hormone) in most peripheral tissues and is essential for the adrenergic receptor mediated thermogenesis in BAT [50]. T3 increases oxygen consumption, which is mediated through PGC-1α and NRF1 [51]. In this study, increased Dio2 mRNA in WATs is speculated to increase local T3 content and further stimulate PGC-1α mRNA expression and the metabolic rate of mice. 

Fucoxanthinol and amarouciaxanthin A are two major metabolites of FX in mouse plasma and liver [52]. In mouse adipose tissue, amarouciaxanthin A is the major metabolite of FX [53]. *In vitro*, amarouciaxanthin A showed a higher activity in suppressing 3T3-L1 adipocyte differentiation than FX, fucoxanthinol and amarouciaxanthin B [54]. The suppression is associated with downregulations of PPARγ and CCAAT-enhancer-binding protein α (C/EBPα) mRNA levels. In the present *in vivo* study, however, PPARγ mRNA expression in eWAT was upregulated by FX, implying that inhibition of adipocyte differentiation might have a minor role on the low WAT mass observed in our *in vivo* study.

### 2.5. FX Increased Mitochondrial Biogenesis and Fusion-Related Gene Expressions in eWAT and iWAT

**Figure 4:**
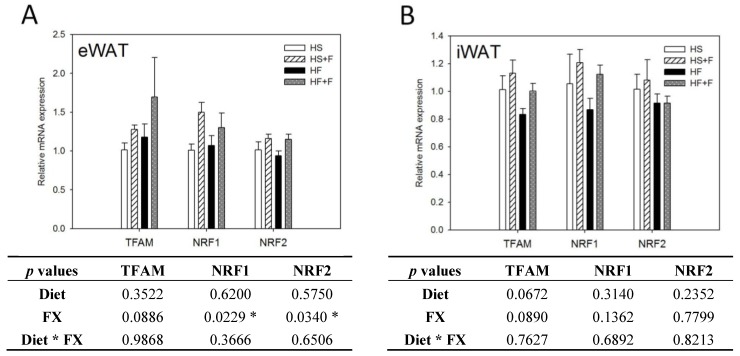
Mitochondrial biogenic gene expressions in epididymal (**A**) and inguinal (**B**) white adipose tissue. Values are means, and error bars are SEM (*n* = 4). * denotes significant effect by either dietary factor at *p* < 0.05 analyzed by two-way ANOVA. HS, HS + F, HF and HF + F: As indicated in Figure 1. The measurement of relative mRNA expression is as indicated in Figure 2.

**Figure 5:**
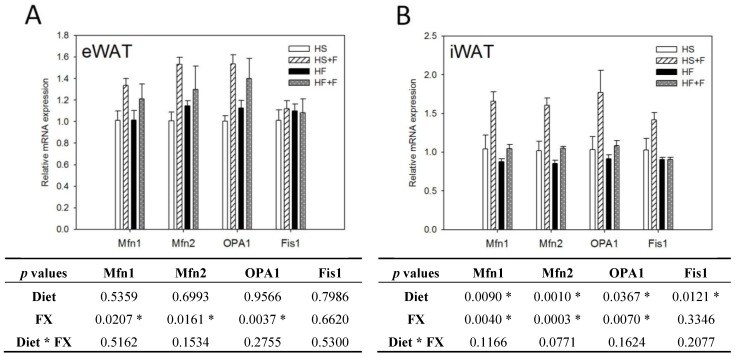
Mitochondrial homeostatic gene expressions in epididymal (**A**) and inguinal (**B**) white adipose tissue. Values are means, and error bars are SEM (*n* = 4). * denotes significant effect by either dietary factor at *p* < 0.05 analyzed by two-way ANOVA. HS, HS + F, HF and HF + F: As indicated in Figure 1. The measurement of relative mRNA expression is as indicated in Figure 2.

FX increased NRF1 and NRF2 mRNA expressions in eWAT, but not iWAT. Diet did not affect TFAM, NRF1 and NRF2 mRNA expressions in eWAT and iWAT (Figure 4). The high fat diet decreased Mfn1, Mfn2, OPA1 and Fis1 mRNA levels in iWAT, but not eWAT. In contrast, FX increased mitochondrial fusion-related genes, including Mfn1, Mfn2 and OPA1, but not fission-related gene Fis1 and mRNA expressions in both eWAT and iWAT (Figure 5). Mitochondria provide ~90% of the cellular energy supply and are also the most important organelle of metabolism. PGC-1α is a strong promoter of mitochondrial biogenesis and oxidative metabolism through NRF1, NRF2, ERRα, PPARγ and PPARα [13,14,15]. PGC-1α targets NRF1 and NRF2 directly or indirectly through ERRα in stimulating nuclear genes required for mitochondrial biogenesis and respiration function [14]. PGC-1α interacts with PPARα in the transcription control of genes encoding mitochondrial fatty acid oxidation enzymes [55]. PGC-1α acts as a partner of PPARγ in the induction of adaptive thermogenesis in brown fat and adipocyte differentiation [14]. The increased metabolic rate of mice fed the FX diets in this study coincides with increased PGC-1α networks. ERRα further activates the transcriptional activity of the Mfn2 promoter, and the effect was synergic with those of PGC-1α [16]. In addition to Mfn2, other mitochondrial fusion-related genes (Mfn1 and OPA1) were also increased by FX in this study. In contrast, mitochondrial fission-related gene Fis1 in WATs was not affected by FX. Mitochondrial fusion is frequently found in metabolically active cells [56], and elevated mitochondrial fusion genes agreed with enhanced VO_2_ and VCO_2_ and the decreased WAT mass of mice in the present study. Among these genes, Mfn2 was shown to be stimulated under cold, adrenergic agonist or exercise treatment through the regulation of PGC-1α and ERRα [16,57]. Therefore, the elevated mitochondrial fusion genes by FX were in accordance with increased PGC-1α and ERRα mRNA in WATs.

### 2.6. Overall Discussion

Using the 2 × 2 factorial design, we demonstrated that irrespective of the diet, FX increased both O_2_ consumption and CO_2_ production, indicating that FX enhanced metabolic rate. This increase in energy expenditure agreed with smaller adipose mass and increases of the PGC-1α network gene expressions in iWAT and eWAT. These included β3-AR, PGC-1α, CIDEA, Dio2, PPARα and genes regulating mitochondria biogenesis and fusion (ERRα, NRF1, NRF2, Mfn1, Mfn2 and OPA1). We speculate that dietary FX might increase the energy expenditure through changes in mitochondria biogenesis, homeostasis and/or activity mediated by the PGC-1α network in eWAT and iWAT.

PGC-1α is a master regulator of energy metabolism that orchestrates cellular responses to various types of metabolic stress, such as fasting, cold temperature and physical exertion. The expression of PGC-1α is induced by such factors as β3-AR and Dio2. It in turn activates the expressions of transcription factors, including PPARs, NRF1/2, ERRα and Mfn2. In this study, FX upregulated PGC-1α and both of its upstream and downstream genes in iWAT and eWAT, implying that the “PGC-1α network” is upregulated. As this network is known to regulate energy metabolism, it coincides with our observation that FX increased energy expenditure.

As we only measured the whole body O_2_ consumption, it is difficult to specify the contribution of different tissues by FX. However, comparison of changes in the gene expression pattern in WATs and BAT prompt us to speculate that WAT might contribute more than BAT in promoting O_2_ consumption.

Moreover, our data together with data of previous reports did not support a major role of BAT in the FX enhanced oxygen consumption. These include: (1) FX downregulated β3-AR, Mfn2, Fis1, Prdm16 and PPARδ mRNA levels in the BAT and did not change other thermogenic genes in this study; (2) some studies [31,38] found an anti-obesity effect of FX without BAT enlargement; (3) oxygen consumption did not correlate to BAT mass in our data (Supplementary Information, Figure S1); (4) FX lowered both serum NE (norepinephrine) and BAT β3-AR expression, which are key regulators of BAT adaptive thermogenesis. Lowered serum NE might be related to a decreased sympathetic activity or an increased NE degradation. It is not known why and how FX lowered serum NE and downregulated BAT β3-AR expression. Although it might not be applicable to FX, there has been a study showing that beta-carotene suppresses exhaustive exercise-induced plasma levels of adrenocorticotropic hormone, norepinephrine and epinephrine by inhibiting the secretion of corticotropin-releasing hormone [58]. Although NE concentration is very important for the activation of PGC-1alpha networks in whole body, factors other than NE, such as PPARs and thyroid receptor activation, *etc.*, can also lead to the activation of the PGC-1α network.

In this study, FX did not significantly increase UCP1 mRNA expression in iWAT, eWAT and BAT (*p* > 0.05). In contrast, expressions of CIDEA and the PGC-1α network in eWAT and iWAT were upregulated, although to different extents. These changes were not noted in liver and skeletal muscle. Moreover, differential extents of induction were also observed in other study. For example, mRNA expression levels of adipogenic marker genes (PPARγ, aP2, adiponectin, C/EBPα, FATP1, LPL and UCP2) and thermogenic genes and mitochondrial biogenesis genes (UCP1, PGC-1α, NRF1, TFAM, Prdm16, CIDEA and Elovl3) induced by triiodothyronine (T3) were also to different extents [59].

## 3. Experimental Section

### 3.1. Preparation of FX

FX was isolated from the dried *Hincksia mitchellae* (Harvey) P. C. Silva brown algae. The brown algae, *Hincksia mitchellae* (Harvey) P. C. Silva, was originally collected from the Taiwan coast and characterized. The material used in this study was obtained by cultivation in enriched seawater medium (SWM-III) in the lab. The dried powder was extracted with acetone in a brown bottle for 24 h, filtered (No. 2, Whatman filter paper, Maidstone, Kent, UK) and the solvent removed, with a temperature lower than 35 °C. The crude extracts were separated by using a silica gel flash column chromatography (Geduran^®^ Si 60, 0.040–0.063 mm, Merck, Darmstadt, Germany), eluted with ethyl acetate/*n*-hexane (4.5:5.5, v/v). The red-orange color fraction was collected and passed through a second silica gel column eluted with ethanol/acetone/*n*-hexane (1:19:80, v/v). After removing the solvent, the residue was re-dissolved in acetone/*n*-hexane (45:55, v/v). An equal volume of Millipore-Q water was added, and the mixture was standing at −20 °C for 4 h until a red precipitate was produced. The precipitate was filtered and dried in a freeze drier. The obtained solid was confirmed as FX by H-NMR and the purity checked by HPLC (PU-980 pump and UV-visible absorbance detector, Jasco, Japan and LiChrospher^®^ 100 RP-18 column, 5 μm, Merck, Darmstadt, Germany). The mobile phase used was methanol/H_2_O (90:10, v/v) with a flow rate of 1 mL/min. The FX was detected at 450 nm and quantified from the peak area by using a standard curve with previous purified and identified FX (95%). The purity of FX prepared was >95% by this HPLC analysis.

### 3.2. Animals and Diets

The animal study was approved by the Institution Animal Care and Use Committee of National Taiwan University (No.NTU-98-EL-106). Three-week old male C57BL/6J mice were purchased from the National Laboratory Animal Center (Taipei, Taiwan) and housed individually in stainless steel wire cages in an animal room with a 12-hr light, 12-hr dark cycle (light period: 0800–2000) and constant temperature (22 ± 2 °C). Mice were fed a non-purified diet (LabDiet^®^ 5001, PMI^®^ Nutrition International Inc., Brentwood, MO, USA) for 1 week and switched to the high sucrose (HS, Table 1) diet for another week before the experiment. After the 2-week acclimation, mice were randomly assigned into four groups (4 mice/group) and respectively fed: HS (50% sucrose, 7% soybean oil), HF (high-fat diet, 23% butter plus 7% soybean oil), HS + F (HS diet supplemented with 0.2% FX) or HF + F (HF diet supplemented with 0.2% FX) test diets for 5 weeks. The composition of the 4 test diets was modified from AIN-93G [37] and our previous studies [60,61] (Table 1). The amounts of casein, cellulose, vitamin and mineral mixtures in the high-fat diets were adjusted to make the nutrient/energy ratios equivalent to those of the HS diet. Mice had free access to the diet and water. Body weight and food intake were recorded weekly.

### 3.3. Metabolic Rate Measurement

After feeding test diets for 2 weeks, mice were placed in the respiratory chamber individually (AccuScan Instruments, Inc., Columbus, OH, USA) with free access to their respective diet food and water. After a 24-h acclimation, mice were continuously monitored in the metabolic chambers for 6 days. Gas samples were collected every minute per mouse, and the data were averaged for each hour. Output parameters from the average of 6 days included the O_2_ consumption (VO_2_), CO_2_ production (VCO_2_) and respiratory quotients (RQ = VO_2_/VCO_2_).

### 3.4. Tissue and Serum Collection

After feeding test diets for 5 weeks, mice were deprived of diet for 12 h and killed by CO_2_ asphyxiation. Blood was withdrawn through retro-orbital. Serum was obtained after centrifugation at 12,000 rpm for 10 min at 4 °C and stored at −80 °C. Liver, spleen, kidney, heart, lung, gastrocnemius muscle, retroperitoneal WAT (rWAT), eWAT, inguinal (iWAT) and intra-scapular BAT were excised, weighed and immediately frozen in liquid nitrogen and stored at −80 °C for mRNA analysis.

### 3.5. Housekeeping Genes PCR Array

rWAT and BAT total RNA (0.5 μg) of 2 mice per group were reversed transcribed using the RT^2^ First Strand Kit and further analyzed by the Mouse Housekeeping Genes PCR array (Qiagen, Germantown, MD, USA), which analyzed 12 commonly used housekeeping genes in a 96-well plate. Briefly, 1 μL cDNA (1 ng/μL), 12.5 μL RT^2^ SYBR Green Mastermix and 11.25 μL water were loaded into primer-precoated wells, and PCR was performed using a StepOnePlus Real-Time PCR System (Applied Biosystems, Foster City, CA, USA). Data were analyzed by three popular algorithms: GeNorm [62], NormFinder [63] and BestKeeper [64]. β-actin was shown to be the most stable and suitable reference gene in our study.

### 3.6. RNA Extraction and Quantitative Real-Time RT-PCR

Total RNA of eWAT, rWAT, iWAT and BAT were isolated by an RNeasy^®^ Plus Universal Mini Kit (Qiagen, Stockach, Germany), according to the manufacturer’s instruction. Total RNA (2 μg) was reverse transcribed to cDNA using a High-Capacity cDNA Reverse Transcription kit (Applied Biosystems, Foster City, CA, USA). PCR was performed in a final volume of 25 μL containing 10 μL cDNA (1 ng/μL), 12.5 μL TaqMan^®^ Gene Expression Master Mix, 1.25 μL probe/primer assay mix and 1.25 μL water. PCR primers and probes were purchased from Applied Biosystems: UCP1, CIDEA (cell death-inducing DFFA-like effecter a), Prdm16 (PR domain containing 16), PGC-1α, NRF1, NRF2, TFAM, ERRα, PPARα, PPARγ, HSL (hormone-sensitive lipase), β3-AR (β3-adrenergic receptor), Dio2 (deiodinase 2), Mfn1, Mfn2, OPA1 (optic atrophy 1), Fis1 (fission 1) and β-actin. These genes are associated with thermogenesis, mitochondrial biogenesis, browning of WAT and mitochondrial fusion and fission. The mRNA expression levels were determined by quantitative real-time RT-PCR using the StepOnePlus Real-Time PCR System (Applied Biosystems, Foster City, CA, USA). The expression levels were normalized to β-actin. Data were analyzed by StepOne Software (v2.2.2, Applied Biosystems, Foster City, CA, USA).

### 3.7. Serum Hormone Analysis

Commercially available ELISA kits were used to measure serum thyroxine (T4) and triiodothyronine (T3) (Calbiotech, Spring Valley, CA, USA), norepinephrine and epinephrine (Labor Diagnostika Nord, Nordhorn, Germany), as well as corticosterone (Cayman chemical company, Ann Arbor, MI, USA).

### 3.8. Statistical Analysis

Data are expressed as the means ± SD or SEM. Statistical analysis were performed using SAS 9.1 (SAS Institute Inc., Cary, NC, USA). For all statistical analyses, data were transformed logarithmically if the variances were non-homogeneous. In order to examine the effect of diet, FX and their interaction in this 2 × 2 factorial design, two-way ANOVAs were used. The effect is considered significant if *p* < 0.05. When an interaction (*p* < 0.05) existed between diet and FX, data were further analyzed by one-way ANOVA and Duncan’s multiple range test. In the metabolic rate study, differences of VO_2_, VCO_2_ and RQ at each time point between HS and HS + F and between HF and HF + F were respectively analyzed by the Student’s *t*-test. Areas under curve (AUCs) of VO_2_ and VCO_2_ were analyzed by two-way ANOVA. The AUC of RQ was analyzed by the Wilcoxon rank-sum test. 

## 4. Conclusions

In conclusion, we demonstrated that dietary FX elevated the metabolic rate and reduced both subcutaneous and abdominal WAT mass irrespective of the diet used. These were associated with upregulated genes of the PGC-1α network, including those regulating mitochondrial biogenesis and fusion in iWAT and eWAT.

## Supplementary Files

Supplementary File 1 Supplementary Information (PDF, 170 KB)

## Abbreviations

DFFA: DNA fragmentation factor, alpha subunit
HF: High fat diet
HF + F: High fat diet supplemented with fucoxanthin
HS: High sucrose diet
HS + F: High sucrose diet supplemented with fucoxanthin
qRT: Quantitative real-time
RQ: Respiratory quotient
