# Supplementary material for: Dietary Fucoxanthin Increases Metabolic Rate and Upregulated mRNA Expressions of the PGC-1alpha Network, Mitochondrial Biogenesis and Fusion Genes in White Adipose Tissues of Mice
Source: Mar Drugs. 2014 Feb 14;12(2):964–82. doi: 10.3390/md12020964 (PMC3944525; doi:10.3390/md12020964)
Supplement: Supplementary File 1 — Supplementary Information (PDF, 170 KB) [file marinedrugs-12-00964-s001.pdf]

Supplementary Information

Table S1. Relative tissue weights of C57BL/6J male mice fed test diets for five weeks. Values are the means ± SD (n = 4). * denotes significant influence by dietary factor at $p < 0.05$ analyzed by two-way ANOVA. HS: High sucrose diet group; HS + F: HS diet supplemented with 0.2% fucoxanthin group; HF: High-fat diet group; HF + F: HF diet supplemented with 0.2% fucoxanthin group.

g/100 g body weight	HS	HS + F	HF	HF + F	*p* values		
					Diet	FX	Diet * FX
Liver	3.96 ± 0.24	5.22 ± 0.29	3.74 ± 0.30	4.89 ± 0.13	0.0474 *	<0.0001 *	0.6596
Kidney	1.40 ± 0.04	1.67 ± 0.14	1.48 ± 0.10	1.54 ± 0.05	0.5932	0.0039 *	0.0472 *
Spleen	0.28 ± 0.02	0.33 ± 0.05	0.28 ± 0.02	0.32 ± 0.02	0.8407	0.0083 *	0.7391
Heart	0.52 ± 0.06	0.66 ± 0.05	0.50 ± 0.03	0.66 ± 0.08	0.7292	0.0003 *	0.7823
Lung	0.65 ± 0.03	0.67 ± 0.08	0.69 ± 0.03	0.70 ± 0.04	0.2163	0.4469	0.8780
gastrocnemius muscle	0.38 ± 0.03	0.41 ± 0.03	0.39 ± 0.02	0.42 ± 0.05	0.7005	0.1335	0.9749

Figure S1. Correlation between fat pad mass and oxygen consumption or carbon dioxide production through the dark period. Data were analyzed by Pearson's Correlation. * denotes significant correlation, $p < 0.05$. HS: High sucrose diet group; HS + F: HS diet supplemented with 0.2% fucoxanthin group; HF: High-fat diet group; HF + F: HF diet supplemented with 0.2% fucoxanthin group.

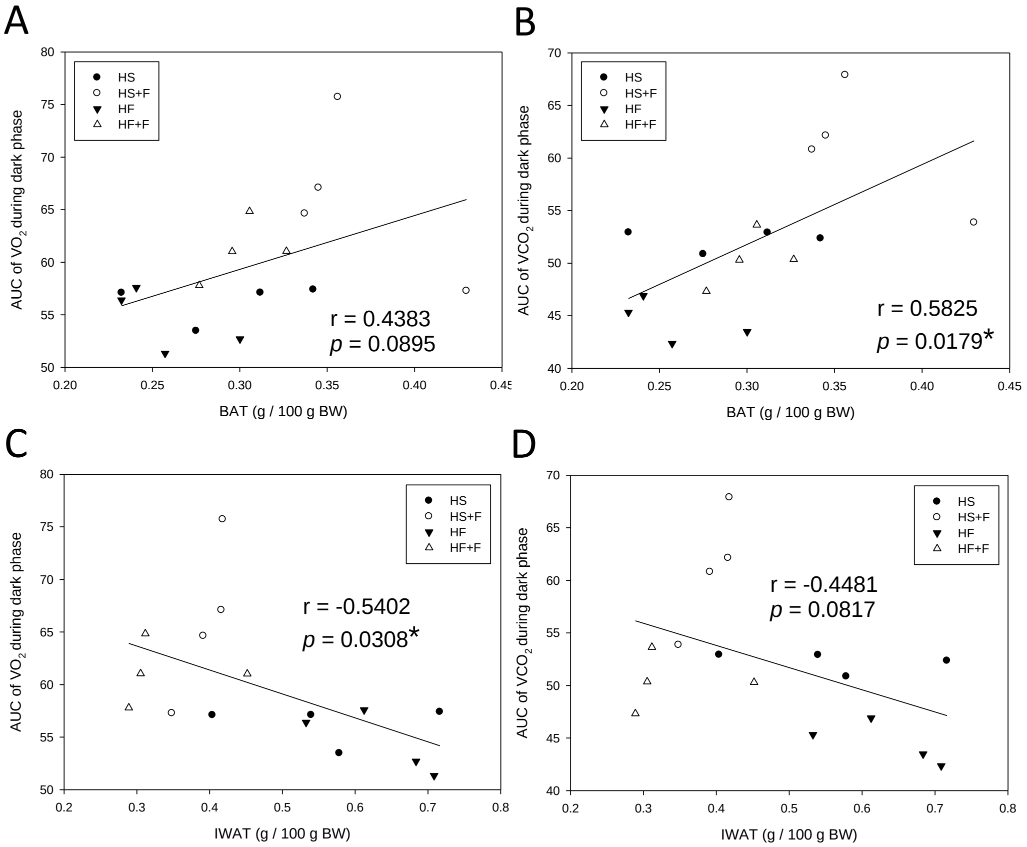

Figure S1. *Cont.*

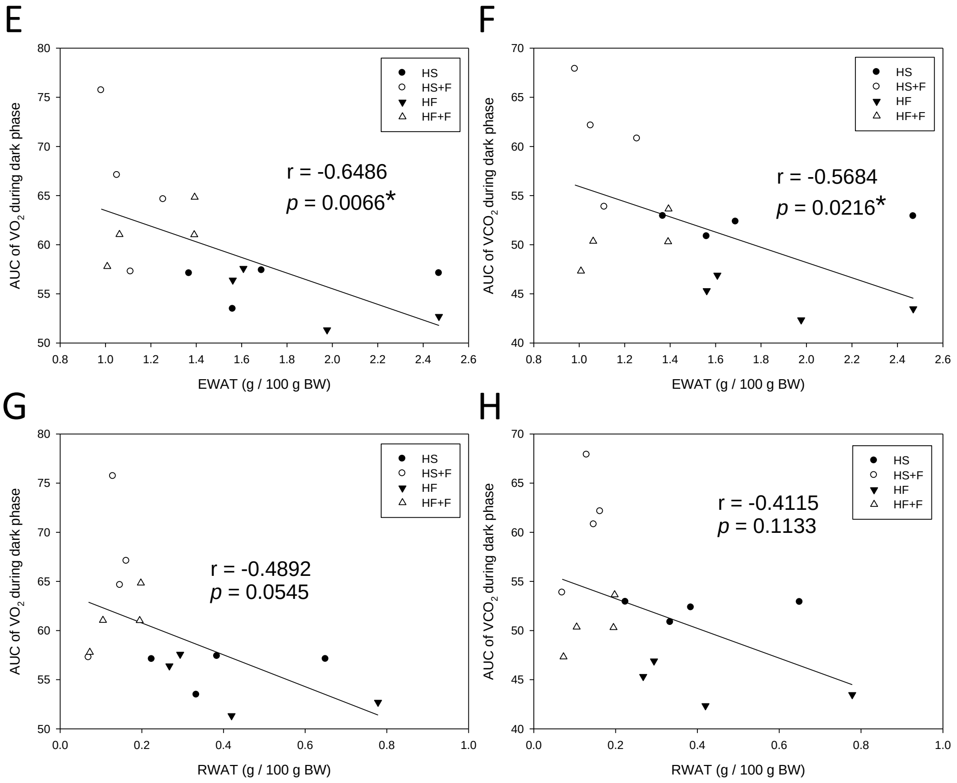

S1. Reduction of Adipose Mass, But No Change in Body Weight

Despite significantly decreased adipose mass in FX-fed mice, the body weight of these mice were not changed. This might be related to enlarged liver, heart and spleen in these mice (Table S1). Enlarged liver and spleen have also been observed in ICR mice orally administered with FX (500 or 1000 mg/kg body weight) daily for 30 days. However, no abnormal changes were observed in histological examination [1]. The dose of FX used in our study, 0.2% in the diet, is equivalent to 180~260 mg/kg body weight per day and is much lower than that study.

S2. Correlation of Adipose Mass and O_2 Consumption and CO_2 Production

As shown in Figure S1, the O_2 consumption during the dark phase of the four groups of mice negatively correlated to the relative weight of eWAT and iWAT, but not rWAT. The CO_2 production, but not O_2 consumption, in the dark phase positively correlated to BAT mass. The CO_2 production in the dark phase negatively correlated to the relative weight of eWAT, but not to those of iWAT and rWAT.

Reference

1. Beppu, F.; Niwano, Y.; Tsukui, T.; Hosokawa, M.; Miyashita, K. Single and repeated oral dose toxicity study of fucoxanthin (FX), a marine carotenoid, in mice. *J. Toxicol. Sci.* **2009**, *34*, 501–510.

© 2014 by the authors; licensee MDPI, Basel, Switzerland. This article is an open access article distributed under the terms and conditions of the Creative Commons Attribution license (http://creativecommons.org/licenses/by/3.0/).
